# Methodologies used in cost-effectiveness models for evaluating treatments in major depressive disorder: a systematic review

**DOI:** 10.1186/1478-7547-10-1

**Published:** 2012-02-01

**Authors:** Evelina A Zimovetz, Sorrel E Wolowacz, Peter M Classi, Julie Birt

**Affiliations:** 1 RTI Health Solutions, The Pavilion, Towers Business Park, Wilmslow Road, Didsbury, Manchester, M20 2LS, UK; 2 Eli Lilly and Company, Global Health Outcomes, Lilly Corporate Center, Indianapolis, IN 46285 USA

**Keywords:** Systematic review, cost-effectiveness analysis, decision analysis, major depressive disorder

## Abstract

**Background:**

Decision makers in many jurisdictions use cost-effectiveness estimates as an aid for selecting interventions with an appropriate balance between health benefits and costs. This systematic literature review aims to provide an overview of published cost-effectiveness models in major depressive disorder (MDD) with a focus on the methods employed. Key components of the identified models are discussed and any challenges in developing models are highlighted.

**Methods:**

A systematic literature search was performed to identify all primary model-based economic evaluations of MDD interventions indexed in MEDLINE, the Cochrane Library, EMBASE, EconLit, and PsycINFO between January 2000 and May 2010.

**Results:**

A total of 37 studies were included in the review. These studies predominantly evaluated antidepressant medications. The analyses were performed across a broad set of countries. The majority of models were decision-trees; eight were Markov models. Most models had a time horizon of less than 1 year. The majority of analyses took a payer perspective. Clinical input data were obtained from pooled placebo-controlled comparative trials, single head-to-head trials, or meta-analyses. The majority of studies (24 of 37) used treatment success or symptom-free days as main outcomes, 14 studies incorporated health state utilities, and 2 used disability-adjusted life-years. A few models (14 of 37) incorporated probabilities and costs associated with suicide and/or suicide attempts. Two models examined the cost-effectiveness of second-line treatment in patients who had failed to respond to initial therapy. Resource use data used in the models were obtained mostly from expert opinion. All studies, with the exception of one, explored parameter uncertainty.

**Conclusions:**

The review identified several model input data gaps, including utility values in partial responders, efficacy of second-line treatments, and resource utilisation estimates obtained from relevant, high-quality studies. It highlighted the differences in outcome measures among the trials of MDD interventions, which can lead to difficulty in performing indirect comparisons, and the inconsistencies in definitions of health states used in the clinical trials and those used in utility studies. Clinical outcomes contributed to the uncertainty in cost-effectiveness estimates to a greater degree than costs or utility weights.

## Introduction

Major depressive disorder (MDD) is a highly prevalent condition estimated to affect 2.3% of the global population [1]. MDD is associated with decreased patient well-being [2], significant burden on health care costs, and productivity losses [3]. It is projected that by the year 2020, depression will rank second in disease burden measured by disability-adjusted life-years [4]. The most common and generally accepted treatment options for patients with MDD include pharmacotherapy, psychotherapy, and pharmacotherapy in combination with psychotherapy [5]. A variety of pharmacotherapies exist for treating MDD; traditionally, these fall into pharmacological classes, such as tricyclic antidepressants (TCAs), tetracyclic antidepressants (non-selective serotonin and norepinephrine reuptake inhibitors), selective serotonin reuptake inhibitors (SSRIs), selective norepinephrine reuptake inhibitors (NRIs), selective serotonin and norepinephrine reuptake inhibitors (SNRIs), monoamine oxidase inhibitors (MAOIs) (including irreversible MAOIs and reversible inhibitors of monoamine oxidase A [RIMAs]), agonists of the melatonin receptor (MT agonists), and other antidepressants [6]. In addition, a wide range of psychotherapeutic options are available, including behavioural therapy, interpersonal therapy, cognitive behavioural therapy (CBT), and the cognitive behavioural analysis system of psychotherapy [6].

With limitations on health care spending, it is important to allocate resources to interventions that are seen to maximise cost-effectiveness. Evaluating the cost-effectiveness of alternative treatment options in MDD can shape policies concerning formulary coverage and reimbursement. A significant number of models evaluating the cost-effectiveness of alternative MDD strategies have been developed. Some of these were examined by Barrett and colleagues [7] in their systematic review of published economic evaluations of interventions for depression. However, no systematic review of the decision-analytic models in MDD has been published recently.

The objectives of this systematic review were to identify published decision-analytic models evaluating the cost-effectiveness of pharmacological treatments in MDD; to examine the variation and frequency of methods employed, highlighting advantages and disadvantages in these methodologies; and to identify specific areas in the MDD cost-effectiveness literature that merited further research to allow improvement in the quality of the economic evaluations.

## Methods

A systematic literature search was performed to identify relevant articles with abstracts indexed in MEDLINE, the Cochrane Library, EMBASE, EconLit, and PsycINFO. A search strategy was developed for each electronic database using a combination of Medical Subject Heading (MeSH) and free-text terms, grouped into the following categories: disease, interventions, economics, and study type. MeSH terms used were 'Depressive Disorder, Major', 'Drug Therapy', 'Antidepressive Agents', 'Costs and Cost Analysis', 'Cost-Benefit Analysis', 'Economics, Hospital', 'Economics, Medical', 'Economics, Nursing', 'Economics, Pharmaceutical', 'Fees and Charges', 'Health Resources/utilization'. The search was limited to articles published in the English language from January 2000 to May 2010. The search strategies were developed by an information specialist with input from authors; full details can be made available on request. Reference lists of identified review articles were checked for relevant studies.

Predefined inclusion criteria were used to determine the selection of the studies. The studies of interest included model-based economic evaluations of pharmacological interventions in MDD (e.g., Markov models, decision-tree models, and models based on mathematical equations). Excluded studies were reviews, editorials, resource use and cost studies, and economic evaluations alongside a clinical trial or those evaluating non-pharmacological interventions only. Study inclusion was performed by the authors and disagreements were resolved by consensus. Data extraction included author and year, country of analysis, analysis type and model structure, analysis time horizon and perspective, treatment comparators, primary outcomes, definitions of effectiveness, sources of primary clinical data, sources of utility data, treatment of uncertainty, and main findings. For each eligible study, data of interest were extracted by one researcher. To ensure quality and accuracy of the data, a second researcher verified the extracted data with their original sources.

## Results

### Search Results and Study Characteristics

**Figure 1:**
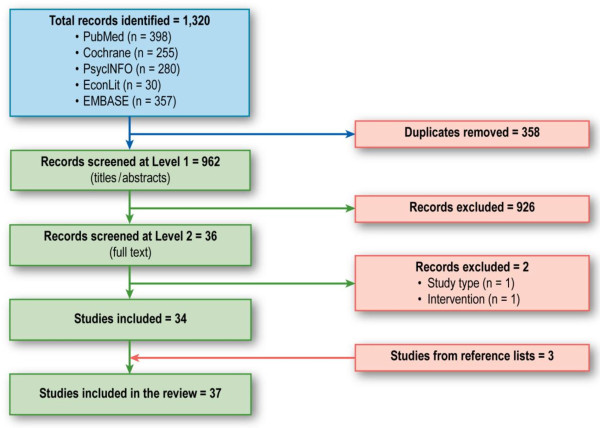
**Flow diagram for selection of studies**.

The search retrieved 1320 citations, 34 of which met the study inclusion criteria, and a further 3 were identified through screening of the reference lists (Figure 1). The characteristics of the included economic models are presented in Table 1[8-45]. Of the 37 included studies, 23 were cost-effectiveness analyses and 14 were cost-utility analyses. The majority of the models (28 of 37) had a decision-tree structure, eight described themselves as Markov models [9-11,16,26,32,33,37], and one [31] did not explicitly report the model structure.

**Table 1:** Summary of methods and conclusions of included studies

Author, Year, Country	Compara-tors	Analysis Type, Model Structure	Time Horizon, Perspective	Primary Out-come	Definition of Effective-ness	Source of Primary Clinical Data	Sensitivity Analyses	Main Finding
Armstrong et al., 2007 [8] US	Escitalopram vs. sertraline	CUA; decision- tree	6 months, payer perspective	QALYs	Response: ≥ 50% improve-ment in MADRS	8-week head-to-head trial	Univariate, probabilistic	Escitalopram dominated^a ^sertraline

Armstrong et al., 2008 [9] US	Escitalopram vs. duloxetine	CUA; Markov structure; 1-week cycle	1 year, payer perspective	QALWs	Remission: MADRS ≤ 12 or HAMD-17 ≤ 7	Pooled analysis of 10 RCTs	Univariate, probabilistic	Escitalopram dominated^a ^duloxetine

Aziz et al., 2005 [10] US	MPT vs. MECT^b^	CUA; Markov structure; 6-month cycle	Lifetime, payer and societal perspective	QALYs	Remission: not explicitly defined	Published literature	Univariate	MECT may be more cost-effective vs. MPT

Benedict et al., 2010 [11] Scotland	Primary care: duloxetine vs. SSRIs, venlafaxine ER, mirtazapine	CUA; Markov structure; 8-week cycle	48 weeks, payer perspective	QALYs	Remission and response: HAMD-17 scale, scores not reported	Pooled analysis of 8 RCTs, a meta- analysis^c^	Univariate, probabilistic	For the commonly accepted WTP thresholds, duloxetine was the preferred option
								
	Secondary care: duloxetine vs. venlafaxine ER, mirtazapine					Pooled analysis of 2 head-to-head trials, a meta-analysis^d^		Duloxetine dominated^a ^venlafaxine ER and mirtazapine

Borghi and Guest, 2000 [12] UK	Mirtazapine vs. amitriptyline	CEA; decision-tree	7 months, payer perspective, societal perspective	Treatment success	Remission: HAMD-17 ≤ 7	Meta-analysis of 4 RCTs of 7-month duration	Univariate	Mirtazapine was cost-effective vs. amitriptyline and fluoxetine
					
	Mirtazapine vs. fluoxetine		6 months, payer perspective, societal perspective	Treatment success	Response: ≥ 50% improve-ment in 17-HAMD	6-week head-to-head trial		

Brown et al., 2000 [13] France	Mirtazapine vs. fluoxetine	CEA; decision-tree	6 months, societal perspective	Treatment success	Response: ≥ 50% improve-ment in 17-HAMD, HAMD-21, or a score of 1 or 2 on CGI	6-week head-to-head trial	Univariate	Mirtazapine was cost-effective vs. fluoxetine

Casciano et al., 2001 [14] 10 countries^e^	Venlafaxine ER vs. SSRIs, TCAs	CEA; decision-tree	6 months, payer perspective	Treatment success, SFDs^f^	Response: 50% improve-ment in HAMD or MADRS	Meta-analysis	Univariate, probabilistic	Venlafaxine ER dominated^a ^SSRIs and TCAs in 9 of the 10 countries

Casciano et al., 2000 [15] US	Venlafaxine ER vs. SSRIs, TCAs	CEA; decision-tree	6 months, payer perspective	Treatment success, SFDs^f^	Response: 50% improve-ment in HAMD or MADRS	Meta-analysis	Univariate, probabilistic	Venlafaxine ER dominated^a ^SSRIs and TCAs

Dardennes et al., 2000 [16] France	Preventative strategy^g ^vs. episodic strategy^h^	CUA; Markov structure; 8-week cycle	12 months, payer perspective	QALYs	Remission: HDRS-21 < 8	12-month double-blind trial	Univariate	Cost of maintenance therapy was partially offset by the gain from recurrence prevention

Demyttenaere et al., 2005 [17] Belgium	Escitalopram vs. citalopram, venlafaxine	CEA; decision-tree	6 months, payer and societal perspec-tives	Treatment success	Remission: MADRS ≤ 12	Meta-analysis of three 8-week RCTs	Univariate, probabilistic	Escitalopram dominated^a ^citalopram and was cost-effective vs. venlafaxine

Doyle et al., 2001 [18] 10 countries^e^	Venlafaxine vs. SSRIs, TCAs	CEA; decision-tree	6 months, payer perspective	Treatment success, SFDs^f^	Response: 50% improve-ment in HAMD or MADRS	2 meta-analyses	Univariate, probabilistic	Venlafaxine dominated^a ^SSRIs and TCAs in 9 of 10 countries (inpatients); 8 of 10 countries (outpatients)

Francois et al., 2002 [19] Finland	Escitalopram vs. citalopram, fluoxetine, venlafaxine	CUA; decision-tree	6 months, societal perspective	Treatment success, QALYs	Remission: MADRS ≤ 12	8-week head-to-head trial, indirect comparison	Univariate	Escitalopram dominated^a ^citalopram, fluoxetine and venlafaxine

Francois et al., 2003 [20] Norway	Escitalopram vs. citalopram, fluoxetine, venlafaxine	CEA; decision-tree	6 months, societal perspective	Treatment success	Remission: MADRS ≤ 12	8-week head-to-head trial, indirect comparison	Univariate	Escitalopram dominated^a ^citalopram, fluoxetine and venlafaxine

Freeman et al., 2000 [21] UK	Venlafaxine vs. SSRIs, TCAs	CEA; decision-tree	6 months, payer perspective	Treatment success, SFDs^f^	Response: 50% improve-ment in HAMD or MADRS	Meta-analysis	Univariate, probabilistic	Venlafaxine dominated^a ^SSRIs and TCAs

Haby et al., 2004 [22] Australia	CBT vs. SSRIs	CEA; decision-tree	9 months, payer perspective	DALYs	Multiple outcomes averaged for individual studies	Meta-analysis	Probabilistic	CBT provided by public psychologist was the most cost-effective option

Hemels et al., 2004 [23] Austria	Escitalopram vs. citalopram	CEA; decision-tree	6 months, payer and societal perspec-tives	Treatment success	Remission: MADRS ≤ 12	8-week head-to-head trial	Univariate, probabilistic	Escitalopram dominated^a ^citalopram

Howard and Knight, 2004 [24] Austria	Venlafaxine ER, venlafaxine IR, SSRIs	CEA; decision-tree	16 week, payer perspective	SFDs^f^	Remission: not explicitly reported	Meta-analysis	Probabilistic	Venlafaxine ER was cost-effective vs. venlafaxine IR and SSRIs; SSRIs were least cost-effective

Kongsakon and Bunchapat-tanasakda, 2008 [25] Thailand	Escitalopram vs. fluoxetine, venlafaxine	CEA; decision-tree	6 months, payer and societal perspec-tives	Treatment success	Remission: MADRS ≤ 12	2 meta-analyses^i^	Univariate, probabilistic	Escitalopram dominated^a ^fluoxetine and venlafaxine

Kulp et al., 2005 [26] Germany	Escitalopram vs. venlafaxine ER	CEA; Markov structure; 2-week cycle	70 days, payer perspective	Treatment success	Response: > 50% improve-ment in MADRS Partial response: 25-50% No response: < 25%	8 week head-to-head trial	None	Escitalopram was cost-effective vs. venlafaxine ER

Lenox-Smith et al., 2004 [27] UK	Venlafaxine vs. TCAs, SSRIs (fluoxetine, paroxetine, and fluvoxamine)	CEA; decision-tree	6 months, payer perspective	SFDs^f^	Remission: 17- HAMD ≤ 7 Response: ≥ 50% improve-ment in HAMD-21	Meta-analysis and a single study	Univariate	Venlafaxine dominated^a ^SSRIs and TCAs

Lenox-Smith et al., 2009 [28] UK	Venlafaxine vs. fluoxetine, amitriptyline	CUA; decision-tree	6 months, payer perspective	QALYs	Remission: HAMD-17 ≤ 7 Response: ≥ 50% improve-ment in HAMD-17	Pooled data from 13 clinical trials	Univariate	Venlafaxine dominated^a ^fluoxetine and amitriptyline; fluoxetine dominated^a ^amitriptyline

Löthgren et al., 2004 [29] Sweden	Escitalopram vs. citalopram, venlafaxine	CEA; decision-tree	6 months, payer and societal perspective	Treatment success	Remission: MADRS ≤ 12	Meta-analysis	Univariate, probabilistic	Escitalopram dominated^a ^citalopram and venlafaxine

Machado et al., 2007 [30] Brazil	SNRIs vs. SSRIs, TCAs	CEA; decision-tree	6 months, payer perspective	Treatment success	Remission: score ≤ 7 on HAMD or ≤ 12 on MADRS	Meta-analysis	Univariate, probabilistic	SNRIs dominated^a ^SSRIs and TCAs

Malone, 2007 [31] US	SSRIs, escitalopram, paroxetine CR, sertraline, venlafaxine ER	CEA; structure not explicitly reported	6 months, payer perspective	Treatment success	Response: ≥ 50% improve-ment in HAMD or MADRS Remission: HAMD ≤ 7 or MADRS ≤ 10	Pooled analysis of trials	Univariate, probabilistic	Venlafaxine had the lowest ICER followed by escitalopram and sertraline^j^; paroxetine was dominated^k^

Nuijten, 2001 [32] The Netherlands	Prolongation of antidepressant medication vs. no prolongation	CUA; Markov structure; 8-week cycle	9 months, payer and societal perspec-tives	QALYs, TWD	Not explicitly reported	Published literature	Univariate	Continuation treatment was not cost-effective, unless extended to maintenance

Perlis et al., 2009 [33] US	Test for SSRI responsive-ness vs. no test	CUA; Markov structure; 3-month cycle	3 years, societal perspective	QALYs	Remission: Instrument not explicitly reported	STAR*D trial [34]	Univariate, two-way	The ICER for the genetic test would not be considered cost effective

Sado et al., 2009 [35] Japan	COMBI vs. AD	CUA; decision-tree	12 months, payer and societal perspec-tives	Treatment success, QALYs	No response: HRSD-17 > 6 or HRSD-24 > 8	Meta-analysis of 8 RCTs	Univariate, probabilistic	COMBI was cost-effective

Simon et al., 2006 [36] UK	COMBI vs. AD	CUA; decision-tree	15 months, payer perspective	Treatment success, QALYs	Remission: HRSD-17 ≤ 6 or HRSD-24 ≤ 8	Meta-analysis	Univariate, probabilistic	COMBI was cost-effective

Sobocki et al., 2008 [37] Sweden	Venlafaxine maintenance treatment vs. placebo	CUA; Markov structure; 1-month cycle	2 years, payer and societal perspec-tives	QALYs	Time to recurrence: 17-HAMD > 12 and ≥ 50% improve-ment in 17-HAMD	2-year trial	Univariate, probabilistic	Maintenance treatment with venlafaxine was cost-effective

Sorenson et al., 2007 [38] Denmark	Escitalopram vs. citalopram, venlafaxine ER	CEA; decision-tree	6 months, payer and societal perspec-tives	Treatment success	Remission: MADRS ≤ 12	Meta-analysis	Univariate, probabilistic	Escitalopram dominated^a ^citalopram; similar cost-effectiveness vs. venlafaxine ER

Sullivan et al., 2004 [39] US	Escitalopram, citalopram, fluoxetine, venlafaxine ER, sertraline, paroxetine, paroxetine CR, venlafaxine	CUA; decision-tree	6 months, payer perspective	QALYs	Response: > 50% improve-ment in MADRS (with treatment maintained for ≥ 180 days)	N/A^l^	Univariate, probabilistic	Escitalopram dominated^a ^all treatments^m^

Trivedi et al., 2004 [40] US	Venlafaxine ER vs. SSRIs (fluvoxamine, fluoxetine, paroxetine)	CUA; decision-tree	8 weeks, payer perspective	QADs and DFDs	Response: HAMD < 15 and/or ≥ 50% improve-ment in HAMD. Remission: HAMD ≤ 7 Response without remission: HAMD 8-14	Pooled analysis of 8 RCTs	Probabilistic	Venlafaxine ER was cost-effective vs. SSRIs

van Baardewijk et al., 2005 [41] Canada	Duloxetine vs. venlafaxine ER	CEA; decision-tree	6 months, payer and societal perspec-tives	Treatment success, SFDs	Remission: HAMD ≤ 7 or MADRS ≤ 10	Meta-analysis	Univariate, probabilistic	Venlafaxine ER dominated^a ^duloxetine

Vos et al., 2005 [42] Australia	Listed in footnote^n^	CEA; decision-tree	9 months and 5 years, payer perspective	DALYs	Multiple outcomes	Meta-analyses	Probabilistic	All interventions had a favourable ICER under Australian health service conditions

Wade et al., 2005 [43] UK	Escitalopram vs. citalopram, venlafaxine	CEA; decision-tree	6 months, payer and societal perspective	Treatment success	Remission: MADRS ≤ 12	Meta-analysis	Univariate, probabilistic	Escitalopram dominated^a ^citalopram; similar cost-effectiveness vs. venlafaxine

Wade et al., 2005 [44] UK	Escitalopram vs. citalopram	CEA; decision-tree	6 months, payer and societal perspec-tives	Treatment success	Remission: MADRS ≤ 12 Response: ≥ 50% improve-ment in MADRS	Meta-analysis	Univariate, probabilistic	Escitalopram dominated^a ^citalopram

Xie et al., 2009 [45] Singapore	Escitalopram vs. venlafaxine, fluvoxamine	CEA; decision-tree	6 months, societal perspective	Treatment success	Remission: MADRS score ≤ 12	Head-to-head trials^o^	Univariate, probabilistic	Escitalopram dominated^a ^venlafaxine and fluvoxamine

AD = antidepressant therapy; CEA = cost-effectiveness analysis; CBT = cognitive behavioural therapy; CGI = Clinical Global Impression; COMBI = combination therapy; CR = controlled release; CUA = cost-utility analysis; DALY = disability-adjusted life-years; DFD = disease-free day; ER = extended release; HAMD = Hamilton Depression Rating Scale; HDRS = Hamilton Depression Rating Scale; HRSD = Hamilton Rating Scale for Depression; ICER = incremental cost-effectiveness ratio; IR = instant release; MADRS = Montgomery Åsberg Depression Rating Scale; MDD = major depressive disorder; MECT = maintenance electroconvulsive therapy; MPT = maintenance pharmacotherapy; N/A = not applicable; QAD = quality-adjusted day; QALW = quality-adjusted life week; QALY = quality-adjusted life-year; RCT = randomised controlled trial; SFD = symptom-free day; SNRI = serotonin-norepinephrine reuptake inhibitor; SSRI = selective serotonin reuptake inhibitor; STAR*D = Sequenced Treatment Alternatives to Relieve Depression trial; TCA = tricyclic antidepressants; TWD = time without depression; UK = United Kingdom; US = United States; WTP = willingness to pay.

^a ^A dominant therapy is less expensive and more effective than the comparator.

^b ^MPT is compared with MECT in elderly individuals with MDD who relapsed after responding to initial course of electroconvulsive therapy.

^c ^Included 6 placebo-controlled RCTs with SSRIs as active arm comparators and 2 head-to-head venlafaxine trials; a meta-analysis was used to obtain response and remission rates for mirtazapine.

^d ^In the absence of efficacy data for mirtazapine in patients with more severe illness, mirtazapine rates were calculated by applying the mean differences between the less severe and the more severe population to the mirtazapine rates reported in the meta-analysis for the patient population with HAMD-17 ≥ 18.

^e ^Germany, Italy, Netherlands, Poland, Spain, Sweden, Switzerland, UK, US, Venezuela.

^f ^SFDs were measured as time elapsed after the determination of success through the end of the period being analysed.

^g ^Continuous treatment with milnacipran.

^h ^Follow-up with no preventive treatment.

^i ^Due to lack of published data on head-to-head comparison of escitalopram and fluoxetine, the clinical data comparing escitalopram to citalopram were used as a proxy, derived from a published meta-analysis.

^j ^ICERs were calculated using SSRIs as the reference group.

^k ^A therapy dominated by other comparators has higher cost and lower effectiveness.

^l ^Due to a lack of consistent and comprehensive studies demonstrating differences in efficacy across all 8 serotonin reuptake inhibitors, and in order to retain a specific focus on the impact of adverse drug reactions on treatment costs, it was assumed that on average 60% of patients respond to serotonin reuptake inhibitor therapy.

^m ^Escitalopram had lowest direct costs and the greatest effectiveness, followed by citalopram, generic fluoxetine, venlafaxine ER, sertraline, generic paroxetine, paroxetine CR, and venlafaxine IR.

^n ^SSRIs (acute, continuation, maintenance), TCAs (acute, continuation, maintenance), Bibliotherapy (acute), Acute and maintenance: individual CBT public psychologist, individual CBT, private psychologist, individual CBT public psychiatrist, individual CBT private psychiatrist, group CBT public psychologist.

^o ^Due to lack of data on head-to-head comparisons of escitalopram and fluvoxamine, a head-to-head comparison of citalopram and fluvoxamine was used as a proxy.

The majority of studies (22 of 37) adopted a 6-month time horizon; most were conducted from the health care payer perspective (19 of 37) and were in adults with MDD receiving first-line therapy (32 of 37).

Of the included studies, 29 examined pharmacological interventions only, four studies evaluated non-pharmacological interventions versus pharmacological therapies [10,16,22,42], two studies included comparisons of pharmacological treatments versus combination therapies (i.e., pharmacotherapy and behavioural therapy) [35,36], one study evaluated the prolongation of pharmacological treatment versus no prolongation following response to initial therapy [32], and one study evaluated pharmacogenetic testing for antidepressant response [33].

The included studies represented 23 countries: Australia [22,42], Austria [23,24], Belgium [17], Brazil [30], Canada [41], Denmark [38], Finland [19], France [13,16], Germany [14,18,26], Italy [14,18], Japan [35], Norway [20], Poland [14,18], Scotland [11], Singapore [45], Spain [14,18], Sweden [14,18,29,37], Switzerland [14,18], Thailand [25], the Netherlands [14,18,32], United Kingdom (UK) [12,14,18,21,27,28,36,43,44], United States (US) [8-10,14,15,18,31,33,39,40], and Venezuela [14,18].

### Modelling Approaches

**Figure 2:**
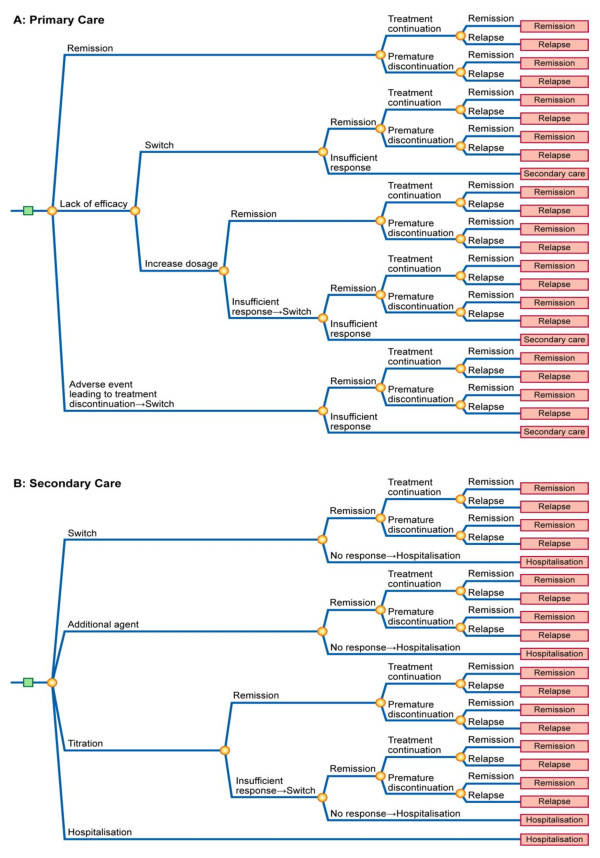
**Two-path model structure reported by Francois et al., 2002**. Reproduced from Francois et al. (2002) [19].

Of the identified models, 28 were described as decision-tree models. Of these, nine [17,19,20,23,25,29,38,43,45] had a similar design based on the model structure reported by Francois [19] (Figure 2). The decision-tree structure used by these models consisted of two paths. Patients with MDD entered the model in the first path representing primary care. Patients with inadequate response in primary care could titrate to a higher dose or switch treatment. Patients with insufficient response after titration and/or switching were referred to secondary care, represented by the second path. In the secondary care path, patients could have their dose titrated, have their treatment switched, receive adjunctive therapy with another agent, or be hospitalised. The model design incorporated the rates of suicide and suicide attempt. Most of the models used the rates of suicide and attempted suicide reported by Khan [46], with two models [19,20] using alternative sources. Three of the studies [25,43,45] that adapted the model structure reported by Francois [19] performed country-specific modifications to better reflect local clinical practice.

**Figure 3:**
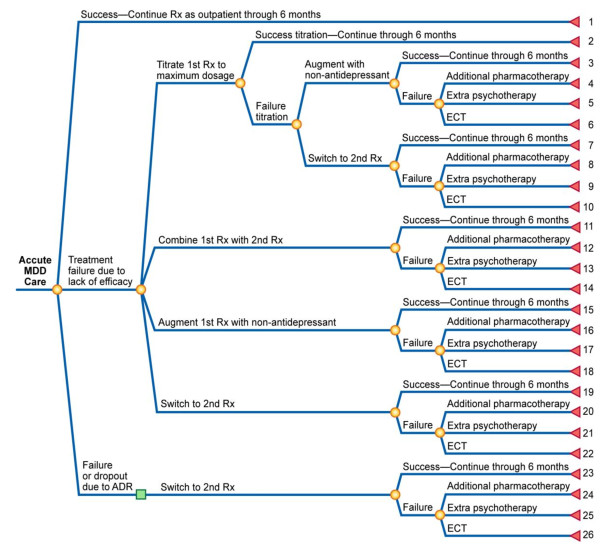
**Model structure reported by Casciano et al., 2000**. ADR = adverse drug reaction; ECT = electroconvulsive therapy; Rx = prescription. Reproduced from Casciano et al. (2000) [15]. Image reprinted with permission from Medscape.com, 2010. Available at: http://www.medscape.com/viewarticle/409930.

Of the decision-tree models identified, six [14,15,18,21,27,28] were based on the structure presented by Casciano [15] (Figure 3). The events modelled following treatment failure due to lack of efficacy included titration to maximum dosage, within-class adjunctive therapy, between-class adjunctive therapy, and treatment switch. Chance nodes for these events were evaluated through consultation with clinical experts. The analysis by Doyle and colleagues [18] covered 10 countries (Germany, Italy, Netherlands, Poland, Spain, Sweden, Switzerland, UK, US, and Venezuela) and performed a clinical management analysis to estimate the country-specific treatment options and outcomes within the set structure of the decision-tree model in Figure 3. Lenox-Smith and colleagues [27,28] amended the structure by including an extra arm representing a clinical outcome node where patient could experience improvement without achieving remission.

The main difference in the model structures reported by Casciano and colleagues [15] and Francois and colleagues [19] is that the latter structure included two paths, one for primary care and one for secondary care. Another difference is that these models incorporated different options for patients failing first and second lines of treatment. The Francois structure offered the option of hospitalisation once all treatment options have been exhausted. For patients experiencing remission, the Casciano structure assumed continuation on treatment for 6 months, whereas the Francois structure incorporated a risk of premature treatment discontinuation. Unlike models based on the structure reported in Francois [19], models adapting the structure by Casciano [15] did not incorporate rates of suicide or suicide attempts.

The systematic review identified 8 models [9-11,16,26,32,33,37] that were described as having a Markov structure. Time horizons ranged from 70 days [26] to lifetime [10], longer than the time horizons in decision-tree models. The cycle lengths of the Markov models ranged from 1 week [9] to 6 months [10]. A variety of health states were defined. In the model by Aziz and colleagues [10], health states were wellness (full remission), partial depression (partial remission), depression (no response), death by suicide, or death by other cause. Benedict and colleagues [11] included relapse, recurrence, and treatment switches. Dardennes and colleagues [16] distinguished between remission with follow-up and remission without follow-up. The model by Perlis and colleagues [33] simply used "depressed" (on or off therapy) and "well" (on or off therapy).

### Model time horizon

**Figure 4:**
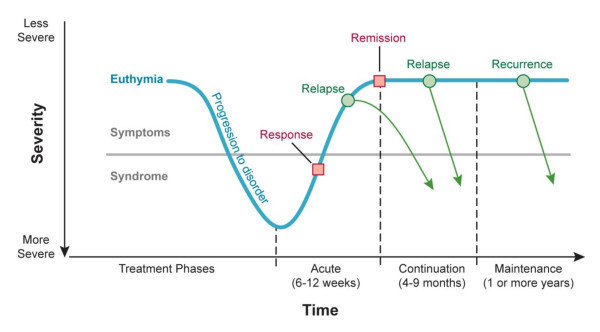
**Phases of treatment for MDD**. MDD = major depressive disorder. Adapted, with permission, from Bakish et al. [49].

A number of economic evaluation guidelines state that the model time horizon is dependent upon the time at which full benefits of the studied intervention can be realised [47,48]. With treatment of depression, certain treatment benefit can be realised over a shorter or a longer time horizon, depending on the treatment stage (Figure 4) [49].

The treatment phases include acute treatment, during which time the goal is to resolve symptoms; continuation treatment, during which time therapy is continued to ensure complete resolution of the index episode and to prevent relapse; and long-term maintenance, during which time optimal therapy is continued to prevent the development of a new episode [50]. Clinical trials in MDD are often conducted over a period of a few weeks, typically 6 to 8 weeks [51], representing the acute phase of a depressive disorder. All three treatment phases should be implemented to optimise treatment outcome [49]. If no improvement is observed after a few weeks of medication, or if undesirable adverse events have occurred, it is recommended to consider titration of the prescribed dose or switch to a different antidepressant. Where improvement is observed, it is recommended to continue the medication until the underlying depression has disappeared [38]. The majority of the identified models had a time horizon of 6 months, covering the acute and continuation phases, or the length of time that patients with a first episode of MDD should normally be treated [27]. Three studies adopted a time horizon of less than 6 months [24,26,40], and three models used a time horizon of 1 year [9,16,35]; other time horizons applied in the identified models included lifetime [10], 3 years [33], 2 years [37], and 15 months [36].

### Patient population

The majority of the models were constructed in a population of adults with MDD who were starting initial therapy, with one study in children and adolescents [22]. No studies were identified in patients with MDD experiencing partial response to initial therapy. Two studies were in patients who did not respond to initial therapy [11,31], two studies were in patients with recurrent depression [16,37], one study was in elderly patients who responded to a course of ECT but then relapsed [10].

### Model comparators

**Figure 5:**
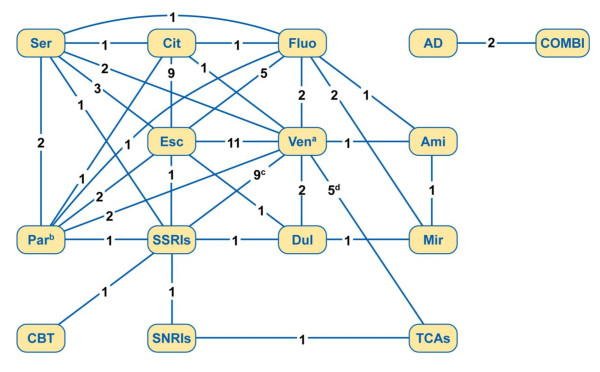
**Diagram of main comparisons included in the review**. AD = antidepressant therapy; ami = amitriptyline; CBT = cognitive behavioral therapy; cit = citalopram; COMBI = combination therapy; Dul = duloxetine; Esc = escitalopram; Fluo = fluoxetine; Fluv = fluvoxamine; Mir = mirtazapine; Par = paroxetine; Ser = sertraline; SNRI = serotonin-norepinephrine reuptake inhibitor; SSRI = selective serotonin reuptake inhibitor; TCAs = tricyclic antidepressants; Ven = venlafaxine. ^a ^Includes both venlafaxine instant release and extended release. ^b ^Includes both generic paroxetine and paroxetine controlled release. ^c ^Of these 9 studies, 2 studies [14,18] compared venlafaxine with SSRIs in outpatient and inpatient settings in 10 countries. ^d ^Of these 5 studies, 2 studies [14,18] compared venlafaxine with TCAs in outpatient and inpatient settings in 10 countries.

The comparisons of MDD treatments evaluated in 30 of the included models focusing on acute and continuation phases are presented in Figure 5. Venlafaxine and escitalopram are the most intensively studied interventions, evaluated in 21 and 15 studies, respectively; followed by SSRIs as a drug class, which were examined in 10 studies, and TCAs as a drug class, which were compared in 6 studies. A number of studies (7 of 37) included treatment evaluations during the maintenance phase. Sobocki and colleagues [37] evaluated venlafaxine maintenance treatment versus placebo. One model [35] compared combination therapy with antidepressant therapy alone. Aziz and colleagues [10] examined maintenance pharmacotherapy versus maintenance ECT. Dardennes and colleagues [16] compared preventative strategy (i.e., a maintenance treatment with milnacipran) and episodic strategy (i.e., medical follow-up treating new episodes when diagnosed). One study [42] included multiple comparisons of treatments in acute and continuation phases (SSRIs, TCAs, CBT, and bibliotherapy) and in maintenance phase (TCAs, SSRIs, and various CBT options). Perlis and colleagues [33] assessed the cost-effectiveness of a pharmacogenetic test for SSRI responsiveness versus a 'no-test' condition. Nuijten [32] assessed the cost-effectiveness of continuation treatment with SSRIs compared with no preventative treatment and examined the impact of extending the continuation treatment to maintenance treatment in a scenario analysis.

### Efficacy and safety data

Both response and remission rates were applied in the economic models as measures of treatment success. However, the definitions of remission and response were not applied consistently across the studies.

Response was most commonly defined as a 50% or greater improvement in the Montgomery Asberg Depression Rating Scale (MADRS) [52] score or the Hamilton Rating Scale for Depression (HAMD-17) score [53]. One study [13] defined response as a 50% or greater improvement in the 21-item HAMD score or a score of 1 or 2 on the patient-rated Clinical Global Impression scale [54]. One study used non-response, defined as a score greater than 6 on the HAMD-17 scale (or greater than 8 on the HAMD-24). One model [26] used three definitions for varying levels of response based on MADRS scale: response (greater than 50% improvement from baseline), partial response (25%-50% improvement from baseline) and no response (< 25% improvement from baseline).

General consensus suggests that values of 7 or less on the HAMD are indicative of clinical remission; for the MADRS instrument, many clinicians have come to accept that values of 10 or less are likely to indicate remission [31]. From the identified models, five studies [9,12,27,28,40] used clinical data that defined remission as a score of 7 or less on HAMD, 10 studies [17,19,20,23,25,29,38,43-45] incorporated remission defined as score of 12 or less on MADRS, two studies [9,30] used both of these definitions, and one study [36] defined remission as a score of 8 or less on the HAMD-24 or a score of 6 or less on the HAMD-17. Out of 14 models using the MADRS-based definition of remission, only two [31,41] used a cut-off value of ≤ 10, with the majority of the models using a cut-off value of ≤ 12.

The variability in the definitions of remission and response applied across the studies presents challenges in performing indirect comparisons of interventions where no head-to-head studies exist. As a result, the majority of studies derived primary clinical inputs from single trials [8,13,16,23,26,33,37,45] or via simple pooling of data from multiple trials [9,28,31,40], or a combination of single trials, pooled analyses, indirect comparisons and meta-analyses [11,12,18-20,25,27]. A fair amount of models (15 of 37) used meta-analyses to obtain primary efficacy inputs [14,15,17,21,22,24,29,30,35,36,38,41-44]. The majority of models (26 of 37) incorporated adverse events; 23 models included adverse events as an outcome leading to treatment discontinuation; only 3 studies [8,9,39] modelled the impact of individual adverse events in terms of utility and costs. Efficacy data applied after change of treatment were most commonly obtained from expert opinion or published literature. Duration of trials used in the models as sources of clinical data were typically between 6 and 12 weeks.

### Resource use and costs

Resource use and cost estimates used in the identified analyses were predominantly obtained and/or validated by expert opinion (22 of 37). Malone and colleagues [31] applied 6-month resource use and costs estimates from a retrospective analysis of accounting records of 1,814 patients enrolled in nine randomised, controlled trials [55]. Sobocki and colleagues [37] used cost data from the naturalistic observational study Health Economic Aspects of Depression in Sweden, conducted in Swedish primary care [56]. For primary care, Wade and colleagues [43] used resource use and cost estimates from the UK General Practice Research Database analysis. Two studies [33,39] used prospective cohort studies in estimating resource use and costs.

The majority of studies employing a payer perspective included medication costs, costs of physicians' time, diagnostic and monitoring tests, hospitalisation and psychotherapy. The specific resources applied varied substantially between the studies. Only a few studies reported the costs of managing adverse events, which were included in the base-case analyses [8,9,12,24,25,39]. A number of studies [10,16,17,19,20,23,25,29,33,43,44] reported the cost of suicide and/or suicide attempt.

Almost half of the identified studies conducted their analyses from the societal perspective. The majority of studies estimated indirect costs associated with productivity losses using the Human Capital approach [11,19,20,23,25,29,38,41,43-45], and a few used the Friction Costs approach [17,32]. The US study in elderly patients with recurrent MDD also included costs associated with lost leisure time activities and wages lost by caregivers [10].

### Health state utilities

Of the identified models, 14 included utility weight estimates to calculate quality-adjusted life-years (QALYs). The utility values applied in the models varied across the same health states by between 0.11 and 0.21, suggesting that the utility weights applied in the models were not consistent (Table 2) [57-68]. Revicki and Wood [62] was the most commonly cited source of utility values applied in the models. This study used the HAMD, SF-36 Health Survey, and standard gamble interviews to obtain utilities for 11 hypothetical depression-related states varying by depression severity (i.e., mild, moderate, severe), medication (i.e., nefazodone, fluoxetine, imipramine), and treatment status (i.e., maintenance treatment or no treatment). The mean utility for severe, untreated depression was the lowest, 0.30. The highest mean utility was for remission without treatment, 0.86. Medication-specific utilities varied from 0.55 to 0.63 for moderate depression, 0.64 to 0.73 for mild depression, and 0.72 to 0.83 for antidepressant remission maintenance therapy [62]. The study by Simon and colleagues [36] used these estimates in the base case analysis, and in the sensitivity analysis the study investigated the effect of partial response to treatment using uncertainty ranges of 0.30 to 0.63 and 0.63 to 0.70 for severe and moderate depression, respectively. The study highlighted the scarcity of evidence on the health-related quality of life of people with depression.

**Table 2:** Health-state utility values applied in published models in MDD

Author, year	Utility value by health state^a^	Primary source and method of utility estimation
Armstrong et al., 2008 [9]; Armstrong et al., 2007 [8]	Treated depression: 0.848 Untreated depression: 0.58	Sullivan et al. [39]: EQ-5D^b^

Aziz et al., 2005 [10]	Pharmacotherapy: Depression: 0.43 Partial depression: 0.55 Well: 0.75 ECT: Depression: 0.52 Partial depression: 0.66 Well: 0.90	Multiple sources: Hatziandreu et al. [57]; Mazumdar et al. [58]; Judd et al. [59]; McDonald et al. [60], Sackett and Torrence [61]

Benedict et al., 2010 [11]	Remitters: 0.79 Responders: 0.68 Non-responders: 0.55 Staying in remission: 0.86	Multiple sources: Eli Lilly, HMBU trial^c ^(data on file): EQ-5D; Revicki and Wood [62]: standard gamble^d^

Dardennes et al., 2000 [16]	Remission with follow-up Preventive strategy: 0.875 Episodic strategy: 0.895 Remission without follow-up: 0.895^e^ Recurrence first 2 months: 0.306^e^ Recurrence months 3 and 4: 0.725^e^ Recurrence months 5 and 6:0.795^e^	Anton and Revicki [63]: standard gamble^f^

Nuijten, 2001 [32]	Depression, on treatment SSRI: 0.70, TCA: 0.64 In remission, treatment prolongation SSRI: 0.80, TCA: 0.72 In remission, off treatment: 0.86 Severe depression: 0.30	Revicki and Wood [62]: standard gamble^d^

Perlis et al., 2009 [33]	Recovered Not on treatment: 0.88 Disutility of treatment: 0.04 Depressed Not on treatment: 0.63 Disutility on treatment: 0.04	Multiple sources: Bennett et al. [64]; Revicki et al. [65]; Revicki and Wood [62]: standard gamble^d^; Schaffer et al. [66]

Sado et al., 2009 [35]	Severe depression: 0.30^g^ Moderate depression: 0.63^g^ Response on treatment: 0.80^g^ Response, no treatment: 0.86^g^	Revicki and Wood [62]: standard gamble^d^

Simon et al., 2006 [36]	Severe depression: 0.30 Moderate depression: 0.63 Remission, treatment: 0.80 Remission, no treatment: 0.86	Revicki and Wood [62]: standard gamble^d^

Sobocki et al., 2008 [37]	Well: 0.86 Episode: 0.57 Remission: 0.81	Sobocki et al. [67]: The study administered EQ-5D questionnaire to 447 patients treated with antidepressant in primary care.

Sullivan et al., 2004 [39]	Treated depression: 0.848 Untreated depression decrement: -0.268 Decrements for ADRs were applied^h^	Sullivan et al. [39]: EQ-5D^b^

ADR = adverse drug reaction; DFD = disease-free day; ECT = electroconvulsive therapy; EQ-5D = the EuroQol Five Dimension instrument; GI = gastrointestinal; MDD = major depressive disorder; MEPS = Medical Expenditure Panel Survey; QAD = quality-adjusted day; SG = standard gamble, SSRI = selective serotonin reuptake inhibitor; TCA = tricyclic antidepressant; UK = United Kingdom.

^a ^Note that some of the studies applied utility values but did not report these explicitly. Francois et al. [19] did not report the utility weights, but stated the source for these as Quality of Life Perspective Study, Lundbeck (data on file). Lenox-Smith et al. [28] transformed DFDs into utility weights using the methodology from Lave et al. [68] using the following assumptions: a non-depressed subject was assumed to have utility score of 1.0 (perfect health); a subject with major depression was assumed to have a utility score of 0.59 (estimated from literature); subjects were assumed to gain 0.41 of QAD for each depression-free day. Trivedi et al. [40] transformed DFDs into utility weights using the methodology from Lave et al. [68], assuming a gain of 0.2 to 0.41 of QAD for each DFD.

^b ^Utility values for each health state were derived from a direct analysis of the data in the 2000 MEPS, which provided individual and variance adjustment weights. The EQ-5D was administered via self-administered questionnaire in MEPS.

^c ^Utility study based on trials (Eli Lilly, HMBU trial, data on file) derived utility values for remitters, responders, and non-responders from EQ-5D scores of European patients using the UK tariffs.

^d ^Utility of remitters staying in remission was obtained from Revicki and Wood [62]. This study used SG method to generate utilities for 11 hypothetical health states. Health states were varied by severity and medication; 70 patients with MDD or dysthymia who completed ≥ 8 weeks of antidepressant treatment were recruited.

^e ^The same values for both episodic strategy and preventative strategy.

^f ^The SG method was used to generate utilities for hypothetical depression-related health states; 70 MDD patients were interviewed cross-sectionally to provide utilities for these health states.

^g ^Both values for combination therapy and antidepressant therapy were assumed to be the same.

^h ^GI: -0.065; Diarrhoea: -0.044; Dyspepsia: -0.086; Nausea: -0.065; Constipation: -0.065; Sexual: -0.049; Excitation: -0.129; Insomnia: -0.129; Anxiety: -0.129; Drowsiness: -0.085; Headache: -0.115; Other (average of all ADRs): -0.085.

Benedict and colleagues [11] used utilities derived from the EuroQol Five Dimension instrument [69] scores of approximately 300 European patients representing the head-to-head clinical trial population. The model used utility values of 0.79 for remitters, 0.68 for responders, 0.55 for non-responders, and 0.53 for those dropping out. Utility of patients achieving remission and staying in remission without treatment (0.86) was obtained from Revicki and Wood [62]. Another UK model [28] used the methodology by Lave and colleagues [68] to transform the disease-free days (DFDs) into utility weights. The number of DFDs between measurements over any given interval was first estimated by adding the calculated number of DFDs for the first and second HAMD-17 scores, dividing by two, and multiplying by the number of days between assessments. The QALYs gained were then estimated assuming a gain of 0.41 of a quality-adjusted day for each whole DFD gained. One model [40] transformed DFDs into quality-adjusted days using utility weights from Lave and colleagues [68].

### Cost-effectiveness results and uncertainty

The findings of the reviewed models are presented in Table 1. The results for the most studied interventions appear to be fairly consistent. Escitalopram was dominant over sertraline (in 2 of 3 analyses [8,39]), duloxetine (in 1 analysis [9]), citalopram (in 9 of 9 analyses [17,19,20,23,29,38,39,43,44]), fluoxetine (in 4 of 4 analyses [19,20,25,39]), fluvoxamine (in 1 analysis [45]), generic and controlled-release paroxetine (in 3 of 3 comparisons [31,39]), and venlafaxine extended release (ER) and instant release (IR) (in 7 of 12 comparisons [19,20,25,29,39,45]). Escitalopram was cost-effective compared with sertraline [31], SSRIs [31], and venlafaxine [17,26]. Escitalopram had similar cost-effectiveness to venlafaxine in two comparisons [38,43] and was less cost-effective in one [31]. Apart from escitalopram, venlafaxine was commonly compared with SSRIs and TCAs and was a dominant strategy in the majority of these comparisons. Among the two studies identified comparing individual SNRIs, duloxetine was dominant over venlafaxine in one comparison [11] and venlafaxine was dominant over duloxetine in the other comparison [41].

All but one study [26] explored parameter uncertainty. Univariate sensitivity analyses were performed in all of these studies. Probabilistic sensitivity analyses were performed in approximately three-quarters of studies. Cost-effectiveness acceptability curves are used in economic analyses to incorporate the joint uncertainty about the effects and costs [70]; these were presented in 5 of 37 studies [8,11,35-37]. Of the 36 studies, 26 conducted comprehensive sensitivity analyses incorporating all important variables. In 14 of these 26 studies, results were not substantially altered in the sensitivity analyses. In 11 studies, varying clinical input parameters impacted the results while in 7 studies, varying resource use or cost parameters changed the results. Of the 26 studies with the comprehensive sensitivity analyses, eight were the cost-utility studies, of which only two showed sensitivity to changes in utility weights.

## Discussion

We reported the main methodological elements of the published decision-analytic models in MDD. The majority of the reviewed models used a decision-tree structure, largely because the analyses explored the acute and continuation phases of depression and relied on clinical inputs from trials of 6 to 12 weeks in duration. Decision-tree models are appropriate for economic modelling in acute illnesses; however, Markov models are the first choice for pharmacoeconomic analysis of chronic diseases like depression [32]. Markov models provide the advantage over decision-tree models by being able to incorporate longer time horizons, which might be more appropriate given the recurrent nature of depression. The fact that the decision-tree structure is commonly preferred over the Markov structure can be explained by the existing data gaps in the clinical evidence necessary to populate a longer horizon multi-state model. A model design combining both the decision-tree and Markov structures could be utilised to accurately capture the short-term trial data for the acute phase and a longer-term events in the maintenance phase.

Only 18 of 37 studies were conducted from the societal perspective. Both utilisation of health care services and productivity losses are high within the MDD patient population, therefore, it is important to consider a broad cost perspective that captures all of the relevant costs to society. Evidence suggests that employment status is more rapidly affected by depression compared with its effect on utilisation of health care services [71,72]. The review of the models suggests that indirect costs have a substantial impact on the outcome of the analysis. In calculation of indirect costs, most models included productivity loss due to absenteeism from work. Productivity loss due to presenteeism (i.e., loss due to patients suffering from symptoms of depression at work resulting in reduced productivity) is also profound [73,74]. Costs associated with lost productivity while at work were examined by one model in the sensitivity analysis [37].

The review identified 14 economic analyses that included QALY as a main outcome. The purpose of QALYs is to provide a value- or preference-based outcome measure incorporating trade-offs between quality of life and quantity of life in a common metric [75]. In recent decades, QALY has become the dominant measure of health value in health technology assessment [76]. Current evidence on the health-related quality of life utilised by the identified models appeared to be scarce, particularly for partial response.

There is a need for further cost-effectiveness studies in patients with MDD who have had partial or no response to the first-line therapy. This review revealed a lack of clinical data in inadequate responders to inform such economic models. More studies focusing on evaluating adjunctive MDD therapies would be welcome. The lack of long-term data describing costs and outcomes substantially limited the reliability of longer-term MDD models.

The review identified some variability in the methods used by the current models, which inevitably makes the interpretation of results more difficult. This variability was seen around a number of methodological domains. First, studies applied different modelling approaches, including both decision-tree and Markov structures. Second, studies utilised different outcomes, with only 14 studies reporting incremental cost-per-QALY estimates. Third, resource use and cost components included in the models varied substantially between the studies, particularly around the resource use assumptions following failure of the initial therapy. This partially reflects differences in health systems; however, also contributing to this is the absence of a large-scale resource utilisation study in MDD in any of the countries covered by the existing analyses. Finally, primary efficacy data used in the models were derived from single trials, pooled analyses, indirect comparisons, meta-analyses, or combinations of these. The variation in methodology for deriving clinical inputs could be explained by the lack of data for some comparators as well as by the difficulty in performing indirect comparisons due to differences in outcome measures between the trials.

Despite some variability in the methods, the results reported by the identified models were broadly consistent. For venlafaxine, a conflicting result was found in two comparisons with duloxetine, with one study reporting venlafaxine ER as a dominant strategy [41] and one study reporting duloxetine as a dominant strategy compared with venlafaxine ER [11]. Given that all other studies reported fairly consistent results for venlafaxine comparisons, this variation could be due to the differences in patient populations between the two studies, with the former study evaluating first-line interventions and the latter study evaluating patients who failed on first-line SSRI. Similarly, another substantial inconsistency was observed in one of 12 comparisons of escitalopram and venlafaxine [31]. Again, this study examined second-line interventions, suggesting that the cost-effectiveness of interventions evaluated as first-line therapies varies substantially compared with the cost-effectiveness of these interventions if used as second-line treatments.

Although model structures varied, overall conclusions regarding the relative cost-effectiveness of interventions were largely consistent. A few exceptions to this were noted. In one study [28], venlafaxine dominated fluoxetine; however, in another study [39], venlafaxine ER was not cost-effective and venlafaxine IR was dominated by fluoxetine. Both models had decision-tree structures, with one [28] using the structure presented by Casciano [15] and the other [39] modelling the initial treatment outcomes in stage one and the treatment of adverse drug reactions in stage two. However, the inconsistency in results also could be due to the use of different measures of response. In comparisons of venlafaxine with SSRIs, one study reported venlafaxine to be dominant [27] while another found venlafaxine to be cost-effective [24]. Both models had decision-tree structures. The longer time horizon in the first study (6 months [27] as opposed to 16 weeks [24]) may have contributed to this difference. Given other parameter differences between these models, it is difficult to conclude with certainty whether or not structural uncertainty is an issue.

The results of the economic models were most sensitive to clinical outcomes than to costs or to utility weights. The uncertainty in key model variables was examined through the use of one-way sensitivity analysis. In approximately half of the models that conducted comprehensive sensitivity analyses, the results were confirmed to be robust. Even if interpreted correctly, however, one-way sensitivity analysis will commonly (in the absence of correlation) underestimate uncertainty, making it particularly vulnerable to false claims that results are robust [77].

Despite the fact that the study by Sullivan and colleagues [39] found that drug-related adverse events have a significant impact on the direct cost and cost-effectiveness of treatments, only three studies considered differences in adverse-event profiles of the individual agents evaluated. Most of the studies modelled discontinuations due to adverse events, as this is equivalent to treatment failure.

Our review had several limitations. Firstly, it did not appraise the quality of the included studies but focused on reporting the methods and data sources used in the models. The second limitation is that we restricted our review to the published literature, therefore excluding searches of the grey literature. Nonetheless, we believe our work provides a comprehensive review of economic models in MDD and could serve as a useful reference for researchers.

## Conclusions

Our review indicated that over the last 10 years a considerable number of economic models have been developed to evaluate the cost and benefits of the interventions for the treatment of MDD. The identified models varied somewhat in their methodology, but the results seemed broadly consistent. In terms of the model input data, the review identified several data gaps, including utility in partial responders, efficacy of second-line treatments, and utilisation estimates obtained from high-quality sources (for example, from observational studies). The review highlighted the difficulty in performing indirect comparisons due to differences in outcome measures between the MDD trials. Achieving consistency with this, and consistency in definitions of health states used in MDD clinical trials and utility studies, would be a large step forward.

## List of abbreviations used in the text

CBT: cognitive behavioural therapy; DFD: disease-free day; ECT: electroconvulsive therapy; ER: extended release; HAMD: Hamilton Rating Scale for Depression; IR: instant release; MADRS: Montgomery Asberg Depression Rating Scale; MAOI: monoamine oxidase inhibitor; MDD: major depressive disorder; MeSH: Medical Subject Heading; MT agonist: agonists of the melatonin receptor; NRI: selective norepinephrine reuptake inhibitor; QALY: quality-adjusted life-year; RIMA: reversible inhibitor of monoamine oxidase A; SNRI: selective serotonin and norepinephrine reuptake inhibitor; SSRI: selective serotonin reuptake inhibitor; TCA: tricyclic antidepressant; UK: United Kingdom; US: United States.

## Competing interests

This study was funded by Eli Lilly and Company. EAZ and SEW served as paid consultants to Eli Lilly and Company and are employees of RTI Health Solutions. PMC and JB are full-time employees and minor shareholders of Eli Lilly and Company.

## Authors' contributions

All authors contributed to the study design and coordination. EAZ performed the literature review and led development of the manuscript. SEW contributed to manuscript development. PMC and JB provided an overall direction and critically reviewed the manuscript. All authors have read and approved the final manuscript.
